# Brain structural correlates of functional capacity in first-episode psychosis

**DOI:** 10.1038/s41598-020-73553-8

**Published:** 2020-10-14

**Authors:** Erkan Alkan, Geoff Davies, Kathy Greenwood, Simon L. Evans

**Affiliations:** 1 Faculty of Health and Medical Sciences, University of Surrey, Guildford, GU2 7XH Surrey UK; 2 School of Psychology, University of Sussex, Brighton, UK; 3 Sussex Partnership NHS Foundation Trust, Sussex, UK; 4 Brighton and Sussex Medical School, Sussex, UK

**Keywords:** Human behaviour, Cognitive neuroscience, Psychosis, Schizophrenia

## Abstract

Impaired functional capacity is a core feature of schizophrenia and presents even in first-episode psychosis (FEP) patients. Impairments in daily functioning tend to persist despite antipsychotic therapy but their neural basis is less clear. Previous studies suggest that volume loss in frontal cortex might be an important contributor, but findings are inconsistent. We aimed to comprehensively investigate the brain structural correlates of functional capacity in FEP using MRI and a reliable objective measure of functioning [University of California, San Diego Performance-Based Skills Assessment (UPSA)]. In a sample of FEP (n = 39) and a well-matched control group (n = 21), we measured cortical thickness, gray matter volume, and white matter tract integrity (fractional anisotropy, FA) within brain regions implicated by previous work. The FEP group had thinner cortex in various frontal regions and fusiform, and reduced FA in inferior longitudinal fasciculus (ILF). In FEP, poorer functional capacity correlated with reduced superior frontal volume and lower FA in left ILF. Importantly, frontal brain volumes and integrity of the ILF were identified as the structural correlates of functional capacity in FEP, controlling for other relevant factors. These findings enhance mechanistic understanding of functional capacity deficits in schizophrenia by specifying the underlying neural correlates. In future, this could help inform intervention strategies.

## Introduction

Impaired functioning is a core feature of psychosis: individuals diagnosed with schizophrenia have poor long-term outcomes in social^1^ and vocational^2^ functioning. Functional disability affects the overwhelming majority of schizophrenia patients and is present at all stages of the illness^2, 3^. However, antipsychotic therapy has limited efficacy in improving functional capacity^4^ with symptomatic remission much more frequently observed than functional recovery^2^. Evidence suggests that functional impairment is linked to negative symptoms in particular: negative symptom severity explains substantial variance in functional capacity both at baseline^5,6^ and over the longer-term^7^, in first episode psychosis (FEP).

However, the underlying neurobiology of functional impairment is not well understood, since relatively few studies have set out with a primary aim of investigating this in individuals with psychosis. A meta-analysis covering both FEP and chronic schizophrenia concluded that prefrontal cortex volume was the most consistent structural correlate of functional capacity although other regions (including limbic structures and ventricular enlargement) were also implicated^8^. Considerable inconsistency in findings was noted, likely at least partially attributable to the heterogeneity of measures used and the fact that most studies explored the question only as a secondary or supplementary analysis. Some used the University of California, San Diego Performance-Based Skills Assessment (UPSA), which assesses functional capacity for everyday life via a series of role-play simulations testing financial, transportation, household chores, communication, and planning skills^9^. Other studies assessed functioning via clinician-rated measures such as the Global Assessment of Function (GAF) scale^10^ or the Camberbell Assessment of Need (CAN) scale. Although well-used and less time-consuming than the UPSA, these measures tend to be less reliable and show lower validity in terms of measuring real-world outcomes^11^. Others used subjective ratings of functional capacity, or more limited proxy measures such as socio-economic status (SES) (e.g. Takayanagi et al.^12^). Using comprehensive objective measures such as UPSA is crucial: it is the preferred methodology as it does not rely on patients’ self-insight^13^. However, the brain structural correlates of UPSA performance has to date only been investigated in schizophrenia, not FEP^14^. The current study addresses this shortcoming in the literature.

In FEP, left prefrontal cortex volume has been shown to predict GAF-rated functional capacity^15^ and social/occupational functioning 1 year subsequent^16^. In ultra-high risk individuals, lower baseline volume in middle and inferior frontal gyrus predicted a subsequent 9-year decline in GAF-rated functioning^17^, while in schizophrenia, cortical thickness in middle/superior frontal has been linked to role functioning ability^18^. Prefrontal cortex functional activation patterns correlate with both cognitive performance and global functioning in FEP^19^, suggesting that prefrontal volume loss might impact functional capacity as a result of executive function impairment. Other studies have linked ventricular enlargement to poorer functional outcome in FEP measured using the CAN scale^20,21^. In chronic schizophrenia, excessive ventricular enlargement has also been associated with poorer functional outcome^22^. Volume in neighbouring regions also shows associations. Right insula volume has been linked to GAF-rated functional decline in FEP^23^ and at-risk individuals^24^. Uwatoko, et al.^25^ found that right insula volume correlated with occupational functioning which was assessed by the instrumental role category score of the Quality of Life Scale (QLS), in schizophrenia. They also reported that this relationship was mediated by negative symptoms. In a study which only focussed on subcortical structures, volume of the right thalamus correlated with UPSA scores in schizophrenia patients^14^. Overall, evidence suggests that volume loss in prefrontal regions, ventricular enlargement, and reduced insula and thalamus volume might constitute the neural correlates of functional capacity in schizophrenia, although not all findings are consistent. Further, since social functioning is an important component of functional capacity, it might be expected that regions implicated in the processing of social stimuli would also contribute. Studies suggest that gray matter reduction in fusiform gyrus might underlie the poorer facial processing abilities often observed in schizophrenia^26^. Accurate perception of social stimuli is critical for some aspects of functional capacity, but whether volume reduction in fusiform gyrus also contributes to functioning has not been explicitly tested. However, evidence does point to a role for white matter integrity within specific tracts, with fractional anisotropy (FA) being the most commonly used measure in the literature for measuring this. Karlsgodt, et al.^27^ found that FA in inferior longitudinal fasciculus (ILF) predicted subsequent deterioration in social and role functioning in 36 individuals at ultra-high risk of psychosis. In a sample of 30 patients with schizophrenia, Behdinan et al.^28^ found that FA of the ILF and arcuate fasciculus correlated with QLS scores, this relationship was mediated by negative symptoms. Thus, integrity of the ILF might be an important contributor to functional capacity: no studies have examined this in FEP but we address this here. Reduced FA in left ILF has been reported in FEP, however the significance of this is not clear^29^.

Neuroimaging studies of individuals diagnosed with schizophrenia point to specific abnormalities in brain morphology associated with the disease and the aforementioned findings should be considered in this context. Ventricular enlargement is one of the most well-replicated of these and appears to be progressive in chronic patients^30^. Most studies have shown that ventricular enlargement is also detectable in FEP^31,32^ although this is not always replicated, e.g. Berger et al.^33^. In FEP, volumetric differences in other regions have been reported, although the literature is inconsistent. Overall, perhaps the most robust findings have been within fronto-temporal^34,35^ and thalamo-cortical networks^36^, and regarding the latter, it has been proposed that thalamic pathology might be pivotal to the disease due to the role of this structure in coordinating information flow within and between neural networks^37^. In FEP, volumetric decreases in thalamus, insula, and anterior cingulate have been consistently identified^38^. In addition, cortical thickness reductions have been reported, particularly across frontal regions^39–44^ although some studies have also identified thinning in parietal and temporal regions including fusiform gyrus^39,42^. Regarding cortical thinning, the studies that have investigated this in FEP point to a fairly consistent pattern of thinning across frontal and parietal cortices^44^ sometimes accompanied by thinning in temporal regions^42,45^. However, Song et al.^46^ found only thinning in left insula and superior temporal gyrus.

In this study, we set out to investigate the brain structural correlates of functional capacity in FEP using UPSA, an optimal objective measure of functioning. Firstly, we used multiple regression to determine which demographic and symptom severity variables influenced UPSA scores: given previous findings, we expected negative symptoms to explain substantial variance. For the MRI analyses, we focused on volume, cortical thickness, and FA in specific regions of interest (ROIs) implicated by previous work. On these MRI measures, the FEP group was first contrasted with an age-, gender- and education- matched control group so as to contextualise the findings. Then we explored correlations between these measures and UPSA scores in the FEP group, controlling for the factors identified in the multiple regression analysis. This approach allowed us to focus specifically on the neurobiological correlates of functional capacity, independent of other explanatory variables.

## Methods

### Subjects

Ethical approval for the study was given by the London-Camden and Islington NHS Research and Ethics Committee (Ref: 11/LO/1877, project ID 72141). All research was performed in accordance with relevant guidelines and regulations. Written informed consent was obtained on the day of the study. 39 patients with FEP (9 females, age range 19–39, see Table 1) were recruited from Early Intervention in Psychosis services in Sussex Partnership NHS Foundation Trust (requiring duration of untreated psychosis < 2 years). Neuroleptic medication information was taken and converted to olanzapine and chlorpromazine equivalent doses^47^. The short form of the Positive and Negative Syndrome Scale^48^ was applied to assess patient’s symptoms.

**Table 1:** Demographic characteristics and UPSA scores of the sample.

	Healthy control (N = 21)	FEP (N = 39)	*p*
Age, M (SD)	24.43 (5.62)	26.31 (5.57)	0.219^a^
Female (N, %)	5 (23.8%)	9 (23.1%)	0.949^b^
Education (years) (SD)	13.71 (1.76)	13.28 (1.68)	0.355^a^
Medication (olanzapine equivalent mg/day) (SD)	N/A	13.15 (6.92)	N/A
Medication (chlorpromazine equivalent mg/day)	N/A	223.72 (274.56)	N/A
PANSS (3 item) positive symptoms (mean) (SD)	N/A	5.34 (2.40)	N/A
PANSS (2 item) cognitive disorganisation (mean) (SD)	N/A	3.31 (1.45)	N/A
PANSS (3 item) negative symptoms (mean) (SD)	N/A	5.74 (2.80)	N/A
UPSA	N/A	68.41 (19.18)	N/A

*FEP* first-episode psychosis, *M* (*SD*) mean (standard deviation), *PANSS* Positive and Negative Syndrome Scale, *N/A* not applicable.

^a^Independent sample *t* test, ^b^Pearson Chi-square.

A further 21 age, gender, and education -matched healthy controls were also recruited (5 females, age range 18–38). For all participants, inclusion criteria were: age > 18 years, MRI safe, and no history of neurologic illness/substance misuse. FEP participants were required to have a primary diagnosis of FEP, while controls had no history of psychiatric illness. Additional measures were included within the test session and results from these have been reported elsewhere^3, 49–52^. In particular, please refer to Rae et al.^52^ for analyses of microstructural white matter abnormalities in this sample based on NODDI.

### MRI acquisition

All imaging data was acquired with a Siemens Avanto 1.5 T scanner (Siemens Erlangen, Germany) using a 32-channel head coil and gradient strength 44 mT/m. To detect any artefacts, all images were visually inspected prior to analysis.

#### T1 structural

A high-resolution T1-weighted structural image was acquired using an MPRAGE sequence with the following parameters: TR/TE = 2730 ms/3.57 ms; GRAPPA acceleration 2; an in-plane matrix of 256 × 256 pixels over a FOV of 256 mm × 240 mm; flip angle 7°; slice thickness 1 mm yielding 192 sagittal plane slices; coronal and axial resolution 1 mm; acquisition time 5 min 58 s.

#### Gray matter volume and cortical thickness measurements

The gray matter volumes and cortical thickness measures were calculated by FreeSurfer 6.0.0 using the “recon-all” pipeline (https://surfer.nmr.mgh.harvard.edu). Parcellation was based on the Destrieux atlas^53^.

#### Neurite orientation dispersion and density imaging (NODDI)

Three diffusion-weighted shells were acquired using b = 300, 800, and 2400 s/mm^2^ with 9, 30, and 60 unique non-colinear diffusion directions, respectively. In addition, 11 non-diffusion-weighted volumes (i.e. b ≈ 0) were acquired. A NODDI microstructural model was computed and fitted to the data using the NODDI toolbox for MATLAB^54^ that provided measures of neurite density, orientation dispersion, and FA in native space. After normalisation, binarized masks (from the JHU white-matter tractography atlas included with FSL^55^) were applied to calculate FA in the ILF.

Note. Further details regarding the pre-processing and the acquisition of T1 structural, gray matter volume, and cortical thickness measurements are presented in Supplementary Materials.

### The University of California, San Diego Performance-Based Skills Assessment (UPSA)

Functional capacity was measured by the UCSD Performance-based Skills Assessment (UPSA)^9^. The UPSA is an instrument to assess the capacity to complete everyday tasks across 5 domains: finance, communication, comprehension and planning, transportation, and household chores. The UPSA has been assessed for reliability and validated in schizophrenia^56^ and employed in FEP before^3,6,51,57^. Of the 39 FEP participants, 34 completed the UPSA.

### Statistical analysis

IBM SPSS Statistics 21.0 was used to conduct statistical analyses. On demographic variables, groups were compared using independent sample *t* tests (for age and years of education), and a χ^2^ test (for gender).

To identify significant predictors of UPSA in the FEP group, multiple linear regression analysis was performed (enter method): age, gender, years of education, PANNS negative, and chlorpromazine equivalent were entered as independent variables. Multicollinearity was observed to be low: the VIF values never approached or exceeded the generally-accepted critical value of 10, and in fact, were typically much lower (< 3).

For between-group comparisons of cortical thickness, an ROI approach was taken using regions that have been associated with functional capacity by previous studies. Specifically, as indicated by a meta-analysis^8^ prefrontal cortex is most consistency implicated, with previous findings pointing to structural correlates across middle and lateral prefrontal cortex^15–17^: as such we included Superior Frontal, Right VLPFC, Left VLPFC, Middle Frontal, Rostral Anterior Cingulate in our set of ROIs. Previous work has also implicated ventricular enlargement^20–22^, right insula volume^23–25^, and volume of the right thalamus^14^ to poorer functional outcome; thus these regions were also included. We also included fusiform due to its role in the processing of social stimuli social functioning which is an important contributor to functional capacity; despite this, it has not been explored by previous work. For FA measures, we focused only on bilateral ILF as reduced FA in this tract has been consistently implicated in functional capacity by two prior studies^27,28^. Group comparisons of the neural indices were analysed using analyses of covariance (ANCOVAs), with age and gender as covariates.

#### Thickness ROIs

Superior Frontal, Right VLPFC, Left VLPFC, Middle Frontal, Rostral Anterior Cingulate, Right Fusiform, Left Fusiform, Right Insula.

#### Volume ROIs

Superior Frontal, Right VLPFC, Left VLPFC, Middle Frontal, Rostral Anterior Cingulate, Right Fusiform, Left Fusiform, Right Insula, Right Thalamus, Right Lateral Ventricle, Left Lateral Ventricle.

#### FA ROIs

Right ILF, Left ILF.

To investigate the relationships between functional capacity (UPSA) and brain structural measures (volume and thickness within the same ROIs), partial correlations were performed, with age, medication, and PANSS^-^ scores as control variables. To test the relationships between functional capacity (UPSA) and FA in ILF, partial correlations were performed. Previous published work using the NODDI data from these participants^52^ showed that ILF FA was not related to medication or symptom severity, but there was an effect of age. Therefore age was included as a control variable in the FA partial correlation analyses.

## Results

### Demographic data of the study sample

No statistically significant differences were observed between FEP and healthy controls on any demographic variables (all p values > 0.05, see Table 1). In the FEP group, the mean UPSA score was 68.41 (SD = 19.18, N = 34), which is slightly lower than previous FEP studies in which their mean scores were 74.7^51^ and 77.5^6^.

### Demographic and clinical predictors of UPSA

Age, gender, years of education, PANSS negative scores, and chlorpromazine equivalent were used as independent variables in a multiple linear regression model to predict the UPSA scores. Using the enter method, a statistically significant model emerged (*F*_*5,27*_ = 3.343, *p* = 0.018, adjusted *R*^2^ = 0.268). Negative symptom severity (p = 0.035) and medication (p = 0.024) were found to be significant predictors of UPSA scores (Table S1).

### MRI—structural differences between FEP and controls

Between-group differences were assessed using ANCOVA with age and gender as covariates. Bonferroni-corrected thresholds for multiple comparisons were applied and the adjusted alpha values were 0.00625 (i.e., 0.05 divided by 8) for thickness, 0.0045 (i.e., 0.05 divided by 11) for volume and 0.025 for FA (i.e., 0.05 divided by 2) within the preselected ROIs.

#### Cortical thickness

After correction for multiple comparisons, thinner cortices were observed in FEP within superior frontal, middle frontal, right VLPFC, and right fusiform. The results also suggested that FEP had thinner cortices in left VLPFC, left fusiform, and right insula, although these did not survive correction for multiple comparisons. There were no statistically significant differences in rostral anterior cingulate (Table 2).

**Table 2:** Cortical thickness, volumes (expressed as percentage of total intracranial volume) and FA in ILF, group comparisons (ANCOVA, age and gender covariates).

Thickness	Mean thickness		*F*	*p*
	HC	FEP		
Superior frontal	2.76	2.63	10.813**	0.002
Right VLPFC	2.63	2.51	10.604**	0.002
Left VLPFC	2.62	2.51	8.056*	0.006
Middle frontal	2.50	2.39	10.077**	0.002
Rostral anterior cingulate	2.73	2.69	0.212	0.647
Right fusiform	2.72	2.62	9.818**	0.003
Left fusiform	2.70	2.63	5.134*	0.027
Right insula	3.07	2.98	4.291*	0.043

Volumes	Mean volume (%ICV)		*F*	*p*
	HC	FEP		
Superior frontal	1.37	1.32	1.179	0.282
Right VLPFC	0.22	0.22	0.097	0.756
Left VLPFC	0.22	0.21	0.237	0.628
Middle frontal	0.68	0.67	0.057	0.811
Rostral anterior cingulate	0.15	0.15	0.005	0.943
Right fusiform	0.62	0.61	0.448	0.506
Left fusiform	0.64	0.63	0.373	0.544
Right thalamus	0.48	0.46	5.291*	0.025
Right lateral ventricle	0.26	0.41	5.143*	0.027
Left lateral ventricle	0.30	0.43	5.093*	0.028
Right insula	0.43	0.41	1.345	0.251

FA	Mean FA		*F*	*p*
Right ILF	0.4689	0.4446	6.865***	0.011
Left ILF	0.4478	0.4287	6.497***	0.014

*HC* healthy controls, *FEP* first episode of psychosis, *VLPFC* ventrolateral prefrontal cortex, *FA *fractional anisotropy, *ILF* inferior longitudinal fasciculus.

***Significant after Bonferroni’s correction for multiple comparisons at p < 0.025, for FA differences, **Significant after Bonferroni’s correction for multiple comparisons at p < 0.00625, for cortical thickness differences; *Significant at an uncorrected threshold, p < 0.05.

#### Gray matter volume and ventricle differences

Results suggested that FEP had lower volume in right hemisphere thalamus, and larger lateral ventricles (bilaterally). However, these differences were not significant after multiple comparisons. There were no statistically significant differences in the other ROIs (Table 2).

#### FA differences

FEP had reduced FA in the right and left ILF compared to healthy controls; these differences survived correction for multiple comparisons (Bonferroni corrected p < 0.025) (Table 2).

### Correlations between UPSA scores and MRI measures

Partial correlation analyses (controlling for age, medication, and PANNS negative scores) were performed to investigate the relationships between UPSA and MRI measures. As above, a Bonferroni correction for multiple comparisons was employed and the adjusted alpha values were 0.00625 (i.e., 0.05 divided by 8) for thickness, 0.0045 (i.e., 0.05 divided by 11) for volume and 0.025 for FA (i.e., 0.05 divided by 2).

#### Grey matter volume

**Figure 1:**
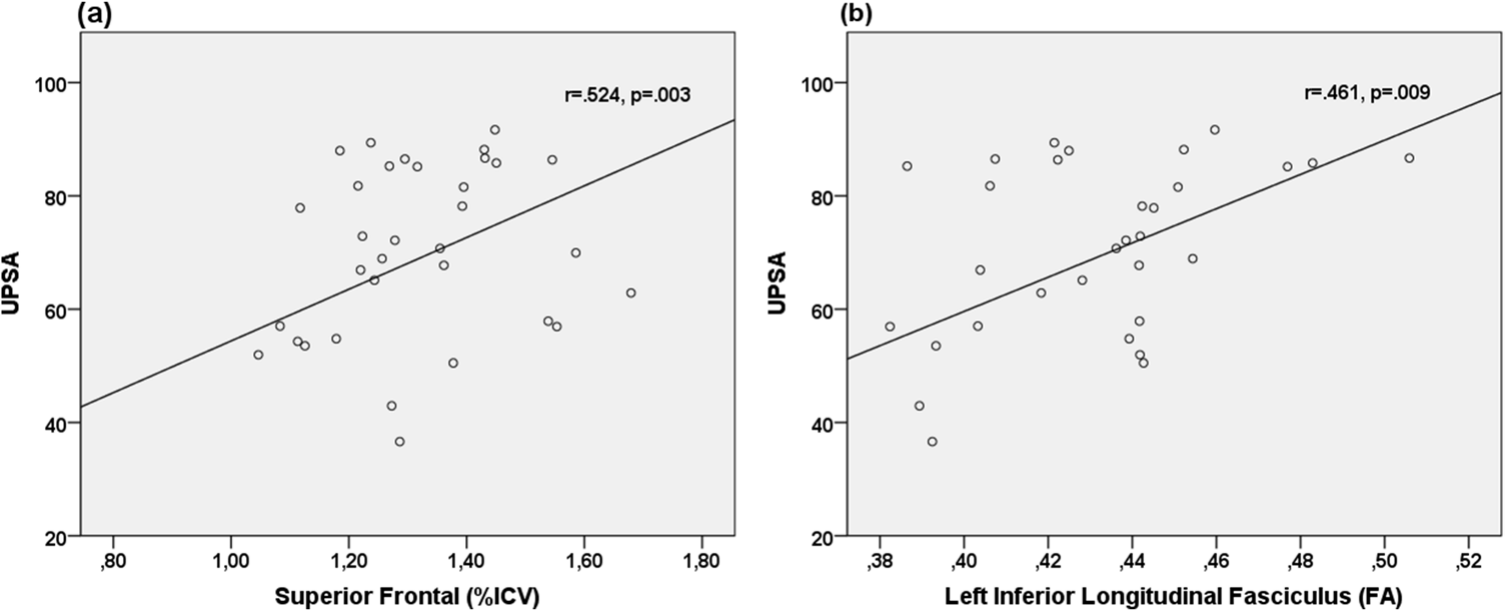
Scatterplots of functional capacity (UPSA) against ICV-adjusted superior frontal volume and FA in left ILF. (**a**) Larger superior frontal volume correlated with higher functional capacity. (**b**) FA in left ILF correlated positively with functional capacity.

UPSA scores in FEP positively correlated with superior frontal volume (see Fig. 1a; Table 3) after correction for multiple comparisons. Associations were also seen in bilateral fusiform, rostral anterior cingulate, right VLPFC, and right insula, but these did not survive correction for multiple comparisons. No associations were seen in the middle frontal, left VLPFC, thalamus, and lateral ventricle (Table 3).

**Table 3:** Brain structural (volumetric) correlates of UPSA (partial correlations, controlling for PANNS^-^, age, and medication). FA (in ILF) correlates of UPSA (partial correlations, controlling for age).

	*r*	*p*
**Volumes**		
Superior frontal	0.524**	0.003
Middle frontal	0.307	0.099
Rostral anterior cingulate	0.445*	0.014
Right VLPFC	0.364*	0.048
Left VLPFC	0.146	0.440
Right fusiform	0.400*	0.028
Left fusiform	0.401*	0.028
Right thalamus	− 0.210	0.266
Right lateral ventricle	− 0.082	0.666
Left lateral ventricle	− 0.175	0.354
Right insula	0.418*	0.021
**FA**		
Right ILF	0.119	0.524
Left ILF	0.461**	0.009

*PANNS*^−^ PANNS negative, *VLPFC* ventrolateral prefrontal cortex, *FA* fractional anisotropy, *ILF* inferior longitudinal fasciculus.

**Significant after Bonferroni’s correction for multiple comparisons at p < 0.0045 for volumes and p < 0.025 for FA; *Significant at an uncorrected threshold, p < 0.05.

#### Cortical thickness correlates

UPSA scores in FEP group did not significantly correlate with thickness in any of the ROIs (all p values > 0.05) (Table S2).

#### FA correlates

UPSA scores in FEP group positively correlated with FA in left ILF, this relationship survived correction for multiple comparisons (see Fig. 1b; Table 3).

## Discussion

Our primary aim was to comprehensively determine the brain structural correlates of functional capacity in FEP. As context, we also analysed brain structural differences between FEP and a healthy control group. In line with previous findings, PANNS negative scores and medication use explained significant variance in UPSA scores. Previous studies suggest negative symptoms are the strongest symptomatic contributor to poor functional capacity in FEP^5,6^ and schizophrenia^58^. Likewise, in the present study negative symptoms emerged as a significant predictor of UPSA scores and underscores the importance of addressing negative symptoms as a treatment goal so as to promote better functioning in FEP.

Relative to the control group, FEP had thinner cortex in superior frontal, VLPFC, middle frontal, and fusiform. This accords with previous studies that point to consistent cortical thinning, particularly in frontal regions^41,42,45,59^. These differences were not apparent when volume rather than cortical thickness was tested, however. Some previous work suggests that cortical thickness might be the more sensitive measure^60^. Cortical thickness reflects the density and size of neuronal cells and neuropil (axons + dendrites + glia)^60,61^ and postmortem studies in schizophrenia point to reductions in these parameters, while the actual number of neurons appears to be unaffected^62,63^. Thus reduced cortical thickness and volumetric indices in schizophrenia are assumed to reflect these alterations^64^.

The volumetric analysis did point to ventricular enlargement and reduced volume in right thalamus in the FEP group, although these did not survive correction for multiple comparisons. Ventricular enlargement in FEP is a common finding^31,32,65^ as is reduced volume in right thalamus^36,38^. We also explored FA in the ILF, based on evidence linking this parameter to functional capacity in patients with schizophrenia^27,28^. Relative to controls, we found FEP to have reduced FA in both right and left ILF, which is in line with the previous findings. Hatton, et al.^29^ and Ashtari, et al.^66^ found reduced FA in the left ILF in young adult FEP patients, suggesting that reduced FA in this tract might be an important neural marker during the early stages of the disease.

The main focus of the current study was to investigate the structural correlates of functional capacity. A reliable objective measure (UPSA) was used and analyses investigated correlations between UPSA scores and brain structural indices within a set of brain structures implicated in previous studies. PANNS negative scores and medication use were controlled for in the analyses. The current paper represents a more thorough investigation compared to previous studies, many of which only investigated the links between structure and functional capacity as a secondary aim, or used a non-optimal measure of functioning^8,11^. A previous study employing UPSA identified a positive relationship between right hemisphere thalamus volume and UPSA scores in patients with schizophrenia^14^ but the current study is the first to comprehensively test brain structural correlates of functional capacity using UPSA in an FEP sample. After correction for multiple comparisons, superior frontal volume was seen to positively correlate with UPSA score (p = 0.003). Positive correlations were also observed with other frontal regions (right superior frontal, right VLPFC, rostral anterior cingulate), although these did not survive correction for multiple comparisons. Positive relationships between functioning and volume in frontal regions have been found previously in FEP^15,16^. Frontal volumetric reduction could lead to cognitive dysfunction and associated difficulties with daily functioning. Links between cognitive deficits and poor real-world functioning are well established in schizophrenia^67,68^. For example, in schizophrenia, links between language ability and UPSA have been identified^69^, correlations between global cognitive performance and UPSA have also been shown^70^ although not necessarily across all UPSA domains^71^. In terms of underlying mechanisms, greater brain surface contraction in the 2 years after first episode of psychosis has been shown in dorsal frontal regions, compared to controls^72^, possibly reflecting an increased rate of synaptic pruning. Here, the fact that UPSA correlations were seen with volume rather than thickness measures is noteworthy. Brain volume is a function of both cortical surface area and thickness. Surface area and thickness appear to be both phenotypically and genetically distinct. Cortical thickness has been shown to be largely uncorrelated with surface area and volume estimates and is influenced by different genetic factors^73,74^. On the other hand, grey matter volume generally correlates with cortical surface area^74^. Investigations in patients with schizophrenia have found regions where grey matter volume reduction is present in the absence of cortical thinning^75–77^ and this might be attributable to surface area changes. Indeed, Kong et al.^78^ identified regions (including superior frontal gyrus) where volume and thickness are discrepant, and subsequent ROI analyses demonstrated that changes in surface area and curvature could account for the discrepancy. Here, UPSA scores correlated with superior frontal volume but not thickness, suggesting that surface parameters in frontal regions could be a factor. Previous studies have found reduced cortical area in patients with schizophrenia (including in parietal, temporal, and medial frontal areas). Rimol et al.^60^ found that while cortical thinning accounted for most of the volumetric differences between patients and controls, it was reduced surface area that explained volumetric differences between schizophrenia and bipolar patients. Analyses of surface area parameters were beyond the scope of the current study, but future work could consider these to investigate the contribution of frontal surface area parameters to functional capacity.

We took a conservative approach to correcting for multiple comparisons (Bonferroni). Although this stringency increases the confidence in our findings, it raises the risk of committing Type 2 errors. Therefore, it worth noting the correlations between UPSA and fusiform (left and right) as well as right insula volumes which did not survive multiple comparisons correction. Right insula volume has been linked to functional capacity in at-risk individuals^24^; FEP^23^ and schizophrenia^25^. This could reflect the putative role of this structure in coordinating information flow within and between neural networks^37^. The possible links between UPSA and fusiform volumes are also interesting. Previous studies on functional capacity have neglected to include fusiform within their choice of ROIs, instead often focusing on frontal structures. The accurate appraisal of social stimuli is important for optimal functioning, therefore it is perhaps to be expected that reduced fusiform volumes, which have been linked to poorer facial processing abilities in schizophrenia^26^, might contribute to functional capacity scores. These correlations merit further exploration in an expanded sample. No links were found here between ventricular enlargement and functional capacity. This does not accord with previous work in FEP^20,21^, although these studies used the CAN scale which focuses on the health and social needs of the patient, whereas UPSA assesses the capacity to complete everyday tasks across several distinct domains.

Finally, we also investigated white matter integrity within the ILF. Lower FA was evident in the FEP group in both right and left ILF. Previous work has identified reduced FA in the left ILF in FEP^29,66^. This was also found by a study in adolescents with schizophrenia^66^, with reduced FA in this tract associated with visual hallucinations. Reductions in right ILF FA have been identified in schizophrenia samples and linked to the severity of positive symptoms^79^ and thinking disorder^80^. Associations between FA in ILF and functional capacity have been identified in ultra-high risk^27^ and schizophrenia^28^ samples but not before studied in FEP. The ILF connects brain regions that are important for social cognition abilities (including fusiform gyrus and amygdala). Indeed, a study in healthy controls found that FA in ILF correlated with facial emotion detection accuracy^81^; studies in other patient populations confirm this relationship^82^. Here, we found FA in left ILF to correlate with UPSA scores in FEP, demonstrating that microstructural abnormalities in this tract have direct consequences for functioning in this patient group. This could be driven by its impact on social cognition ability; future work should explore this possibility. Concerns have been raised regarding the reliability of FA as a marker of white matter integrity, since it is unduly impacted by crossing fibers^83^. Newer, more sensitive measures have been proposed and should be considered in future work^84^.

This study benefits from the use of a matched control group and (unlike many previous studies) the inclusion of a rigorous, objective measure of functioning (UPSA). Although time-consuming, this measure has been suggested to be more reliable and to have higher validity, thus enhancing confidence in our findings^11^. This is the first study to use UPSA in exploring the brain structural correlates of functioning in FEP; also, compared to previous work, a broader range of structural MRI metrics were employed. Study weaknesses include a relatively modest sample size and the lack of a cognitive task battery, which would have allowed the mediating effect of cognitive ability to have been assessed.

In conclusion, this study identified brain structural differences in FEP, including thinner cortex in frontal regions and fusiform gyrus, and reduced FA in ILF. This concurs with previous findings. The consequences of these for functional capacity (UPSA score) were then comprehensively explored and volumetric (rather than cortical thickness) measures in frontal regions showed the strongest correlations. This suggests that, in FEP, loss of surface area in frontal cortex might have important consequences for functional capacity. A possible relationship with fusiform volume was also observed. Further, FA in left ILF showed a strong correlation with UPSA scores, suggesting that abnormalities in this tract, likely impairing connectivity between regions important for social cognition, are also important contributors to poor functional capacity in FEP. Identifying the brain structural correlates of poor functional capacity in FEP points to the neurobiological mechanisms that underlie functional impairment, this line of enquiry is especially important given that functional capacity shows only limited response to antipsychotic treatment.

## Supplementary information

Supplementary Information.

## Data Availability

The datasets generated during the current study are available from the corresponding author on reasonable request.
