# Supplementary material for: Brain structural correlates of functional capacity in first-episode psychosis
Source: Sci Rep. 2020 Oct 14;10:17229. doi: 10.1038/s41598-020-73553-8 (PMC7560620; doi:10.1038/s41598-020-73553-8)
Supplement: Supplementary file 1 — Supplementary Information. [file 41598_2020_73553_MOESM1_ESM.pdf]

Brain structural correlates of functional capacity in first-episode psychosis

Erkan Alkan[1], Geoff Davies[4], Kathy Greenwood[2,3], Simon Evans*[1]

[1] Faculty of Health and Medical Sciences, University of Surrey, Guildford, Surrey, United Kingdom

[2] School of Psychology, University of Sussex, Brighton, United Kingdom

[3] Sussex Partnership NHS Foundation Trust, United Kingdom

[4] Brighton & Sussex Medical School/Sussex Partnership NHS Foundation Trust, United Kingdom

*To whom correspondence should be addressed; Faculty of Health and Medical Sciences, University of Surrey, Guildford, Surrey GU2 7XH, United Kingdom: Tel: +44 (0)1483 686945, email: se0016@surrey.ac.uk

T1 Structural

All images were visually inspected, and then Cortical reconstruction and volumetric segmentation were performed using the FreeSurfer 6.0 image analysis suite (http://surfer.nmr.mgh.harvard.edu). The reconstruction pipeline employed by FreeSurfer includes intensity normalization, motion correction, and the exclusion of non-brain tissue was performed using a hybrid watershed/surface deformation procedure. Images are transformed to Talairach space and the subcortical white matter and deep grey matter structures are segmented [1,2].

Gray Matter Volume and Cortical Thickness Measurements:

Parcellation was based on the Destrieux atlas [3]. Using each participant's T1-weighted MRI, this automated process (which includes skull stripping, intensity normalization, volumetric labelling, surface atlas registration, gyral labelling, and surface extraction) provides automated parcellation of brain regions. The details of procedure have been described elsewhere [4,5]. The gray matter volumes and cortical thickness measures were calculated by FreeSurfer 6.0 using the "recon-all" pipeline (http://surfer.nmr.mgh.harvard.edu). FreeSurfer provides means of regional thickness by calculating the average distance between the pial surface and the white/gray matter boundary for each region [4,5]. The regional volumes were adjusted for total intra-cranial volume (ICV) by dividing the brain regional volume by the ICV and multiplying by 100.

Supplementary Table S1.

Results of Multiple Linear Regression Analysis for Variables Predicting UPSA scores in FEP

	**Unstandardized coefficient**		**Standardized coefficient**		
Variable	***B***	***SE***	***ß***	***t***	***p***
Constant	59.734	29.105		2.052	.050
PANNS Negative	-2.415	1.089	-.368**	-2.218	.035
Chlorpromazine equivalent	-.026	.011	-.378**	-2.386	.024
Age	-.100	.612	-.028	-.164	.871
Gender	-5.353	7.855	-.114	-.681	.501
Education	2.817	2.072	.247	1.359	.185

**p< .05.*

Supplementary Table S2.

Cortical Thickness Correlates of UPSA (Partial correlations, controlling for $PANNS^-$ and age)

**Regions**	***r***	***p***
Superior Frontal	.164	.387
Middle Frontal	.170	.379
Rostral Anterior Cingulate	.257	.178
Right VLPFC	.158	.413
Left VLPFC	.075	.699
Right Fusiform	.144	.456
Left Fusiform	.132	.495
Right Insula	.117	.546

Abbreviations; $PANNS^-$, PANNS Negative: VLPFC, Ventrolateral Prefrontal Cortex

References

1 Dale, A. M., Fischl, B. & Sereno, M. I. Cortical Surface-Based Analysis: I. Segmentation and Surface Reconstruction. *NeuroImage* **9**, 179-194, doi:https://doi.org/10.1006/nimg.1998.0395 (1999).

2 Fischl, B. *et al.* Whole Brain Segmentation: Automated Labeling of Neuroanatomical Structures in the Human Brain. *Neuron* **33**, 341-355, doi:https://doi.org/10.1016/S0896-6273(02)00569-X (2002).

3 Destrieux, C., Fischl, B., Dale, A. M. & Halgren, E. A sulcal depth-based anatomical parcellation of the cerebral cortex. *NeuroImage* **47**, S151, doi:10.1016/s1053-8119(09)71561-7 (2009).

4 Fischl, B. FreeSurfer. *NeuroImage* **62**, 774-781, doi:https://doi.org/10.1016/j.neuroimage.2012.01.021 (2012).

5 Fischl, B., Sereno, M. I. & Dale, A. M. Cortical Surface-Based Analysis: II: Inflation, Flattening, and a Surface-Based Coordinate System. *NeuroImage* **9**, 195-207, doi:https://doi.org/10.1006/nimg.1998.0396 (1999).
